# Wild-Type p53 Protein Enhances APR-246-Induced Cytotoxicity in Acute Myeloid Leukemia and Normal Hematopoietic Stem/Progenitor Cells

**DOI:** 10.3390/ijms27114974

**Published:** 2026-05-30

**Authors:** John B. Cart, David Zhu, Lucas Norris, Sadhna O. Piryani, Li-Chan Chang, Christine E. Eyler, Chang-Lung Lee

**Affiliations:** 1 Department of Radiation Oncology, Duke University School of Medicine, Durham, NC 27708, USA; 2 Department of Pathology, Duke University School of Medicine, Durham, NC 27708, USA; 3 Department of Medicine, Duke University School of Medicine, Durham, NC 27708, USA

**Keywords:** acute myeloid leukemia, tumor suppressor *TP53*, APR-246, hematopoietic stem/progenitor cells

## Abstract

APR-246 (Eprenetapopt) is a small-molecule drug that restores the activity of dysfunctional p53 proteins caused by missense mutations that affect the DNA-binding domain. However, recent studies suggest that APR-246 can also induce cell death in cancer cells that carry wild-type (WT) *TP53.* Here, we aimed to determine the impact of APR-246 on the survival of acute myeloid leukemia (AML) cells using isogenic Molm13 cells that harbor WT *TP53*, a missense mutation of *TP53^R175H^*, or a biallelic deletion of *TP53* (*TP53*^−/−^). Our results showed that Molm13 *TP53*^−/−^ cells were significantly more resistant to APR-246-induced cell death compared with their Molm13 *TP53*^R175H/−^ mutant and Molm13 *TP53^+/+^* counterparts. In addition, knockdown of *TP53* significantly reduced cytotoxicity induced by APR-246 in two *TP53* WT AML cell lines (MV4-11 and OCI-AML2). Moreover, APR-246 markedly decreased the clonogenicity of *TP53* WT hematopoietic stem/progenitor cells (HSPCs) isolated from humans and mice. In contrast, biallelic loss of *TP53*, but not *TP53* missense mutation, significantly increased the resistance of mouse HSPCs to APR-246. Mechanistically, the loss of functional p53 proteins in Molm13 and MV4-11 cells decreased intrinsic apoptosis and impaired the production of cellular reactive oxygen species (ROS) induced by APR-246. Together, our results indicate that, in at least a subset of AML cell lines and normal HSPCs, APR-246-induced ROS production and cytotoxicity are enhanced in the presence of WT p53 proteins.

## 1. Introduction

Acute myeloid leukemia (AML) is a rare cancer of the myeloid lineage, characterized by uncontrolled proliferation of immature myeloid precursor cells in the bone marrow [1]. Instead of developing normally, these cells accumulate in a blast-like state as myeloblasts and overtake healthy blood cell production [2]. This results in typical symptoms of fatigue, recurrent infections, and increased susceptibility to bleeding or bruising. According to Cancer Stat Facts published by the National Cancer Institute, AML predominantly affects older adults, with an incidence of 4.2 cases per 100,000 people annually in the United States. The overall 5-year survival rate of adult AML patients is approximately 30.5% [3]. The prognosis of AML is influenced by mutations in the tumor suppressor *TP53*, which are found in approximately 5–10% of de novo AML patients and include missense and truncating mutations [4,5,6]. The median survival for *TP53* wild-type (WT) AML patients is approximately 18 months, while *TP53*-mutated AML results in an overall median survival of only 6 months [7], independent of age or fitness. Thus, there is an unmet need to develop novel approaches that can effectively induce cell death in both *TP53* WT and *TP53* mutant AML cells to improve the survival of this patient population.

Multiple compounds have been developed to selectively target dysfunctional p53 proteins that carry dominant-negative missense mutations [8,9,10,11]. One such compound is APR-246, which is also known as Eprenetapopt or PRIMA-1MET [12,13,14]. This compound is a prodrug that is metabolized into its active form, methylene quinuclidinone (MQ), which will covalently bind to the free thiol groups of exposed cysteines present in mutant p53 proteins [15,16]. This property allows MQ to initiate conformational changes that restore the WT function of mutant p53 proteins [16,17]. Other studies of APR-246 have shown that it can generate significant amounts of reactive oxygen species (ROS) accumulation in tandem with the ability to restore mutant p53 function. This ROS accumulation subsequently triggers a cascade of programmed cell death that includes ferroptosis or apoptosis [18,19,20,21]. Previous research has shown that this ROS accumulation may be a non-targeted effect of APR-246 binding to thiols outside of the mutant p53 protein—more specifically, the thiol-rich glutathione (GSH) synthesis pathway, which has been shown to play an important role in regulating cellular response to ROS accumulation [18,19,20]. The conversion of APR-246 to MQ enables it to covalently bind to GSH, thioredoxin, and other thiol antioxidants, thereby decreasing the tolerance of cancer cells to ROS buildup [22,23]. Elevated GSH levels are commonly observed in tumor cells as they must combat oxidative stress and detoxify drugs to support tumor progression [24]. Together, these findings reveal the dual function of APR-246 to restore the function of mutant p53 and increase ROS generation in cancer cells.

Although numerous papers have demonstrated the promise of APR-246 in treating *TP53*-mutant myeloid neoplasms in preclinical models [13,25,26] and early-stage clinical trials [27,28,29,30,31,32], the failure to meet the primary endpoint in a Phase III clinical trial (NCT03745716) [33] has led to the discontinuation of developing APR-246 for targeting *TP53*-mutant cancers. Nevertheless, emerging evidence from recent papers has provided encouraging data to support the potential use of APR-246 in treating cancer cells beyond those that contain missense mutations of *TP53* [18,21,34,35]. However, the impact of *TP53* mutational status on the response of AML cells to APR-246 remains incompletely understood. Therefore, we investigated this question utilizing isogenic cells that harbor WT *TP53*, a missense mutation of *TP53^R175H^* (*Trp53^R172H^* in mice)*,* or a biallelic deletion of *TP53* (*TP53*^−/−^) to determine the influence of *TP53* mutational status on the cellular response to APR-246 in human AML cell lines and mouse hematopoietic stem/progenitor cells (HSPCs).

## 2. Results

### 2.1. Loss of p53 Increases the Resistance of Human AML Cells to APR-246

**Figure 1:**
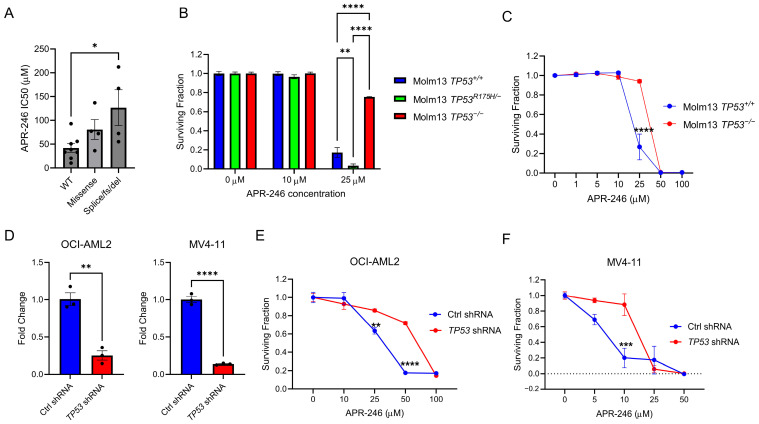
Loss of p53 increases the resistance of human acute myeloid leukemia (AML) cells to APR-246 in vitro. (**A**) Half maximal inhibitory concentration (IC50) of APR-246 in human AML cell lines with various *TP53* mutational status. These AML cells harbor either wild-type (WT) *TP53*, missense mutations of *TP53*, or splicing mutations (splice), frameshift mutations (fs), or deletion (del). The plot was generated by re-analyzing the results published by Birsen et al. Each dot represents one cell line. * *p* < 0.05 by one-way ANOVA with Bonferroni post hoc test. (**B**) Isogenic human Molm13 AML cell lines harboring WT *TP53* (*TP53^+/+^*), a *TP53* R175H missense mutation (*TP53^R175H/−^*), or a biallelic deletion of *TP53* (*TP53^−/−^*) were treated with MES control (0 µM), 10 µM, and 25 µM APR-246 for 72 h in vitro. The surviving fraction was assessed using a resazurin assay. *N* = 3 independent experiments. ** *p* < 0.01 and **** *p* < 0.0001 by two-way ANOVA with Bonferroni post hoc test. (**C**) Isogenic human Molm13 AML cell lines harboring WT *TP53* (*TP53^+/+^*) or a biallelic deletion of *TP53* (*TP53^−/−^*) were treated with a range of 0 to 100 µM APR-246 for 72 h in vitro. The surviving fraction was assessed using a resazurin assay. *N* = 3 independent experiments. **** *p* < 0.0001 by two-way ANOVA with Bonferroni post hoc test. (**D**) Examination of *TP53* mRNA expression in isogenic human OCI-AML2 and MV4-11 AML cell lines expressing control shRNA (Ctrl) or an anti-*TP53* shRNA (*TP53* shRNA). Each dot presents one independent experiment. ** *p* < 0.01 **** *p* < 0.0001 by two tailed *t*-test. (**E**,**F**) Isogenic human OCI-AML2 and MV4-11 AML cell lines expressing control (Ctrl) shRNA or an anti-*TP53* shRNA (*TP53* shRNA) were treated with a range of APR-246 for 72 h in vitro. The surviving fraction was assessed using a resazurin assay. *N* = 3 independent experiments. ** *p* < 0.01 *** *p* < 0.001 **** *p* < 0.0001 by two-way ANOVA with Bonferroni post hoc test. All data are presented as mean ± SEM.

We first conducted a retrospective analysis of published data to examine the half maximal inhibitory concentration (IC50) of APR-246 in human AML cell lines with various *TP53* mutational status, including WT, missense mutations, splicing mutations, frameshift mutations, or deletion [18]. Our analysis revealed that *TP53* WT AML cell lines exhibited a significantly lower IC50 compared with AML cell lines harboring truncated mutations of *TP53* (splicing mutations, frameshift mutations, or deletion) (Figure 1A). We next validated this finding using isogenic human Molm13 AML cell lines that harbor WT *TP53*, a missense mutation of *TP53^R175H^*, or biallelic loss of *TP53* (*TP53*^−/−^) [36]. Results from published data have demonstrated that both Molm13 *TP53^−/−^* and Molm13 *TP53^R175H/−^* cells are substantially more resistant to conventional chemotherapeutics or ionizing radiation compared with Molm13 *TP53^+/+^* cells [36,37]. However, in contrast to the response to genotoxic agents, we observed that APR-246 induced significantly higher cytotoxicity in Molm13 *TP53^+/+^* and Molm13 *TP53^R175H/−^* cells compared with Molm13 *TP53^−/−^* cells at 25 μM (Figure 1B). We conducted additional experiments by treating Molm13 *TP53^+/+^* and Molm13 *TP53^−/−^* cells with a range of APR-246 dosages between 0 and 100 μM and observed a steep dose response of Molm13 cells to APR-246 treatment. While Molm13 *TP53^−/−^* cells were significantly more resistant to APR-246 than Molm13 *TP53^+/+^* cells at 25 μM, 50 μM of APR-246 caused complete cell death in both cell lines (Figure 1C). Collectively, our results using a range of clinically relevant concentrations of APR-246 [32] indicate that Molm13 cells that harbor WT *TP53* or *TP53^R175H^* missense mutation are relatively sensitive to APR-246 compared to Molm13 *TP53^−/−^* cells in a dose-dependent manner.

To validate this observation in additional human *TP53* WT AML cell lines, we generated stable cell lines in which the expression of *TP53* was knocked down in OCI-AML2 and MV4-11 cells (Figure 1D). Our results from the dose–response experiments showed that knockdown of *TP53* significantly decreased cytotoxicity induced by APR-246 in both OCI-AML2 and MV4-11 cell lines (Figure 1E,F). Together, our findings from isogenic human AML cell lines reveal that, despite the difference in baseline sensitivity to APR-246 across Molm13, OCI-AML2, and MV4-11 cells, loss of p53 renders these *TP53* WT cell lines resistant to APR-246 in vitro.

### 2.2. Wild-Type p53 Enhances Apoptosis in Molm13 and MV4-11 Cells Treated with APR-246

**Figure 2:**
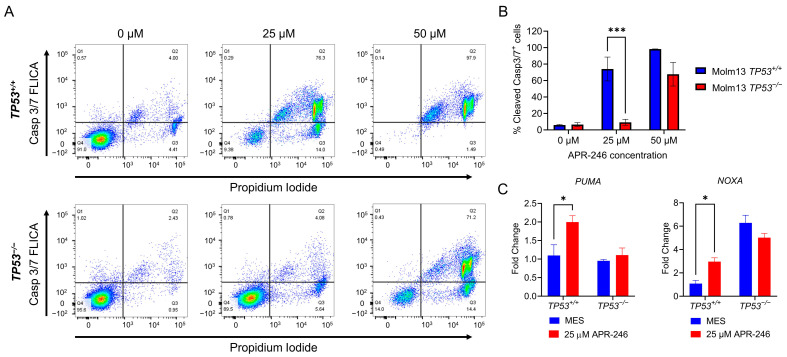
Loss of p53 suppresses APR-246-induced Caspase-3-mediated apoptosis in Molm13 AML in vitro. (**A**) Representative flow cytometry plots of Molm13 *TP53^+/+^* and Molm13 *TP53^−/−^* cells treated with MES control (0 µM), 25 µM, and 50 µM APR-246 for 16 h in vitro. Fresh cells were stained with the FLICA Caspase-3/7 Assay Kit and propidium iodide (PI). The pseudocolor gradient represents cell density, ranging from blue (low density) to red (high density) (**B**) Quantification of cleaved caspase-3/7 positive, including both PI^+^ and PI^−^, in Molm13 *TP53^+/+^* and Molm13 *TP53^−/−^* cells treated with MES control (0 µM), 25 µM, and 50 µM APR-246 for 16 h in vitro. *N* = 3 independent experiments. The data are presented as mean ± SEM. *** *p* < 0.001 by two-way ANOVA with Bonferroni post hoc test. (**C**) Examination of *PUMA* and *NOXA* mRNA in Molm13 *TP53^+/+^* and Molm13 *TP53^−/−^* cells treated with MES control (0 µM) and 25 µM APR-246 for 6 h in vitro. *N* = 3 independent experiments. The data are presented as mean ± SEM. * *p* < 0.05 by two-way ANOVA with Bonferroni post hoc test.

It has been shown that APR-246 can induce various types of cell death in cancer cells, likely in a cell-type-dependent manner [18,19,21]. Thus, we examined the type of cell death induced by APR-246 in Molm13 AML cells by performing a flow cytometry-based FLICA caspase-3/7 assay, which specifically detects cells undergoing the intrinsic pathway of apoptosis (Figure 2A). We observed that 25 μM of APR-246 induced cell death—defined by positive staining for either cleaved caspase-3/7 and/or propidium iodide (PI)—in more than 90% of Molm13 *TP53^+/+^* cells 16 h after treatment, while cell death was detected in less than 20% of Molm13 *TP53^−/−^* cells (Figure 2A and Figure S1). Following 25 μM of APR-246 treatment, Casp3/7^+^ apoptosis was the predominant type of cell death in Molm13 *TP53^+/+^* cells and was significantly suppressed in Molm13 *TP53^−/−^* cells (Figure 2B). Approximately 75% of Molm13 *TP53^+/+^* cells were Casp3/7^+^ 16 h after 25 μM of APR-246 treatment, while only less than 10% of Molm13 *TP53^−/−^* cells were Casp3/7^+^ (Figure 2B). However, 50 μM of APR-246 induced substantial cell death in both Molm13 *TP53^+/+^* and Molm13 *TP53^−/−^* cells, which was also predominantly caused by Casp3/7^+^ apoptosis (Figure 2A,B and Figure S1). RT-qPCR analysis of transcriptional targets of *TP53* at 6 h after 0 μM (MES buffer) or 25 μM of APR-246 treatment revealed significant upregulation of pro-apoptotic genes *PUMA* and *NOXA*—direct transcriptional targets of *TP53* that initiate cell death—in Molm13 *TP53^+/+^* cells, while the induction of these genes was abrogated in Molm13 *TP53^−/−^* cells (Figure 2C). We repeated the RT-qPCR experiment in MV4-11 cells treated with MES buffer or 10 μM of APR-246 for 6 h. Our results showed that *PUMA* and *NOXA* mRNA expression was significantly induced by APR-246 in *TP53* WT cells, but not in cells with *TP53* knockdown (Figure S2). Together, our results indicate that in Molm13 and MV4-11 AML cells, APR-246 primarily induces the intrinsic pathway of apoptosis that is enhanced in the presence of WT p53 protein.

### 2.3. Wild-Type p53 Enhances APR-246-Induced ROS Generation in Molm13 and MV4-11 Cells

**Figure 3:**
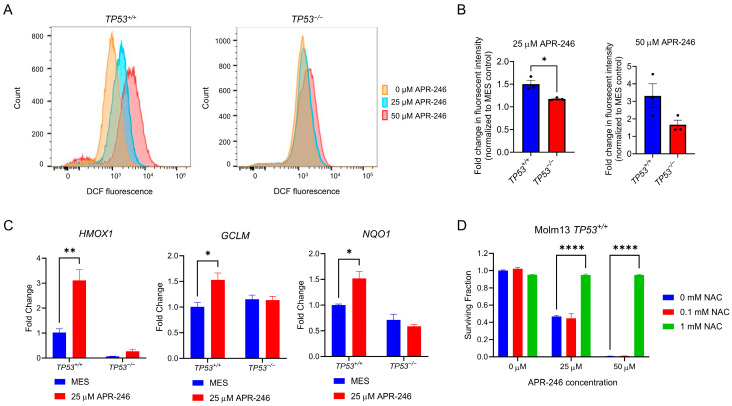
Loss of p53 suppresses the induction of reactive oxygen species by APR-246 in Molm13 AML in vitro. (**A**) Representative flow cytometry plots of Molm13 *TP53^+/+^* and Molm13 *TP53^−/−^* cells treated with MES control (0 µM), 25 µM, and 50 µM APR-246 for 6 h in vitro. Fresh cells were incubated with DCFH-DA, which is a cell-permeant indicator for reactive oxygen species (ROS). DCFH-DA can be hydrolyzed by intracellular esterase to DCFH. When oxidized by the intracellular ROS, DCFH forms fluorescent DCF that can be detected by flow cytometry. (**B**) Quantification of the fold change in the fluorescent intensity of DCF in cells incubated with DCFH-DA. The data are presented as mean ± SEM. * *p* < 0.05 by two-tailed *t*-test. (**C**) Examination of mRNA expression of NRF2 target genes, including *HMOX1*, *GCLM*, and *NQO1* in Molm13 *TP53^+/+^* and Molm13 *TP53^−/−^* cells treated with MES control (0 µM) and 25 µM APR-246 for 6 h in vitro. *N* = 3 independent experiments. The data are presented as mean ± SEM. * *p* < 0.05 and ** *p* < 0.01 by two-way ANOVA with Bonferroni post hoc test. (**D**) Molm13 *TP53^+/+^* cells were treated with MES control (0 µM), 10 µM, and 25 µM APR-246 for 72 h in vitro. 0, 0.1, or 1 mM of N-acetyl cysteine (NAC) was added to the cells immediately after the initiation of APR-246 treatment. The surviving fraction was assessed using a resazurin assay. *N* = 3 independent experiments. The data are presented as mean ± SEM. **** *p* < 0.0001 by two-way ANOVA with Bonferroni post hoc test.

It has been shown that ARR-246 treatment results in the accumulation of ROS by decreasing intracellular levels of cysteine and GSH [18,19,20]. Therefore, we examined the induction of intracellular ROS in Molm13 *TP53^+/+^* and Molm13 *TP53^−/−^* cells treated with 0, 25, and 50 μM of APR-246 for 6 h using the ROS detector 2′,7′-dichlorodihydrofluorescein diacetate (DCFH-DA). DCFH-DA can be hydrolyzed by intracellular esterase to DCFH, which forms fluorescent DCF that can be detected by flow cytometry when oxidized by intracellular ROS [38] (Figure 3A). We observed a dose-dependent increase in ROS generation, as shown by the DCF fluorescent signal, in Molm13 *TP53^+/+^* cells 6 h following APR-246 treatment, while the induction of ROS by APR-246 was markedly reduced in Molm13 *TP53^−/−^* cells (Figure 3A). Quantification of the DCF signal revealed that the loss of WT *TP53* significantly decreased the generation of ROS induced by 25 μM of APR-246 (Figure 3B). To assess the cellular response to APR-246-induced ROS in Molm13 *TP53^+/+^* and Molm13 *TP53^−/−^* cells, we examined NRF2-mediated signaling, which is a key pathway that is activated in response to oxidative stress [39,40]. Our results from RT-qPCR showed that 25 μM of APR-246 significantly induced mRNA expression of three robust NRF2 target genes: *HMOX1*, *GCLM*, and *NQO1* [41] (Figure 3C). In contrast, the induction of these NRF2 target genes by 25 μM of APR-246 was abrogated in Molm13 *TP53^−/−^* cells (Figure 3C). The mRNA expression of *HMOX1*, *GCLM*, and *NQO1* was significantly induced by 10 μM of APR-246 in *TP53* WT MV4-11 cells, but not in MV4-11 cells with *TP53* knockdown (Figure S3). These results indicate that loss-of-function perturbation of p53 reduces ROS accumulation following APR-246 treatment in Molm13 and MV4-11 cells.

To examine whether APR-246 induces cell death of Molm13 cells by targeting the cysteine/ROS axis, we performed rescue experiments using N-acetylcysteine (NAC)—a precursor of L-cysteine that can act as an ROS scavenger [42,43]. We exposed Molm13 *TP53^+/+^* and Molm13 *TP53^−/−^* cells to varying doses of APR-246 (0 μM, 25 μM, and 50 μM), while also treating each cohort with varying concentrations of NAC (0 mM, 0.1 mM, and 1 mM). Our results showed that 1 mM of NAC significantly increased the surviving fraction of Molm13 *TP53^+/+^* and Molm13 *TP53^−/−^* cells treated with 25 μM and 50 μM of APR-246 (Figure 3D and Figure S4). Collectively, our findings reveal that APR-246 induces cell death of Molm13 cells through the cysteine/ROS axis and that the induction of intracellular ROS by APR-246 is enhanced in the presence of WT p53 protein.

### 2.4. Loss of p53 Increases the Resistance of Hematopoietic Stem/Progenitor Cells to APR-246

**Figure 4:**
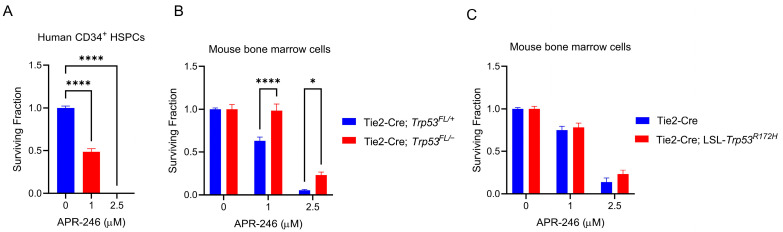
Deletion of p53 increases the resistance of hematopoietic stem/progenitor cells to APR-246 in vitro. (**A**) Human CD34^+^ cord blood cells were plated in methylcellulose-based media and were treated with MES control (0 µM), 10 µM, and 25 µM APR. The surviving fraction was assessed based on the results from the colony-forming cell (CFC) assay. *N* = 3 independent experiments. (**B**) Whole bone marrow cells from Tie2-Cre; *Trp53^FL/+^* and Tie2-Cre; *Trp53^FL/−^* mice were plated in methylcellulose-based media and were treated with MES control (0 µM), 10 µM, and 25 µM APR. The surviving fraction was assessed based on the results from the CFC assay. *N* = 3 independent experiments. (**C**) Whole bone marrow cells from Tie2-Cre and Tie2-Cre; LSL-*Trp53^R172H^* mice were plated in methylcellulose-based media and were treated with MES control (0 µM), 10 µM, and 25 µM APR. The surviving fraction was assessed based on the results from the CFC assay. *N* = 3 independent experiments. The data are presented as mean ± SEM. * *p* < 0.05 and **** *p* < 0.0001 by two-way ANOVA with Bonferroni post hoc test. ns: not significant.

Given the fundamental difference in ROS levels between cancer and normal cells [44], we next examined the role of WT *TP53* in regulating the response of normal HSPCs to APR-246. Our results from the colony-forming cells (CFC) assay revealed that cord blood-derived human CD34^+^ HSPCs were highly sensitive to APR-246. These cells exhibited an almost 50% reduction in clonogenicity at 1 μM of APR-246, with virtually all cells losing clonogenic potential at 2.5 μM (Figure 4A). We then conducted experiments using bone marrow-derived HSPCs isolated from transgenic mouse models where one or both copies of *Trp53* were deleted in hematopoietic cells by Tie2-Cre [37]. We observed that HPSCs that retain one WT allele of *Trp53* (Tie2-Cre; *Trp53^FL/+^*) exhibited a significant decrease in CFC compared with HPSCs that lose both alleles of *Trp53* (Tie2-Cre; *Trp53^FL/−^*) at 1 μM and 2.5 μM of APR-246 (Figure 4B). In addition, we examined the response to APR-246 in mouse HSPCs that harbor WT *Trp53* (Tie2-*Cre*) or a missense mutation of *Trp53^R172H^* (equivalent to human *TP53^R175H^*) (Tie2-Cre; LSL-*Trp53^R172H^*) [37]. When treated with 0 μM (MES), 1 μM, and 2.5 μM APR-246, we observed no significant difference in CFC between the *Trp53* WT and *Trp53^R172H^* HSPCs (Figure 4C). Together, consistent with our findings in human AML cells, our results reveal that the complete loss of p53 increases the resistance to APR-246 in normal HSPCs.

## 3. Discussion

In this study, we elucidate the role of WT p53 protein in regulating the cellular response of AML cells and HSPCs to APR-246 using isogenic human cell lines and genetically engineered mouse models. Biallelic deletion or knockdown of *TP53* substantially increases the resistance to APR-246 in three human AML cell lines (Molm13, OCI-AML2, and MV4-11) compared to their *TP53* WT counterparts (Figure 1). The same p53-dependent increase in the sensitivity to APR-246 is also shown in normal mouse HSPCs (Figure 4). Additionally, this phenomenon is specific to the loss of p53 because Molm13 human AML cells or normal mouse HSPCs harboring a missense mutation of *TP53* are not resistant to APR-246 compared to their *TP53* WT counterparts (Figure 1 and Figure 4). These findings are consistent with the results reported in a recent paper showing that p53 deficiency increases the resistance to APR-246 in pancreatic cancer cells [35] and *Eμ-Myc* lymphoma cells [21]. Collectively, these findings reveal an important role of WT p53 in enhancing the sensitivity to APR-246 in malignant and normal cells.

Our results reveal a dose-dependent response of AML cells and HSPCs to APR-246 in the context of p53 functional status. For Molm13 cells, although 25 μM of APR-246 selectively induces cell death in *TP53^+/+^* cells, treatment with APR-246 at 50 μM causes the death of nearly 100% of *TP53^+/+^* and *TP53^−/−^* cells (Figure 1). We also observe this phenomenon in OCI-AML2 and MV4-11 AML cells, as well as in normal HSPCs (Figure 1 and Figure 4). These findings indicate that while the presence of p53 enhances the sensitivity of AML cells and normal HSPCs to low-dose APR-246, high-dose APR-246 can cause indiscriminate death of these cells through p53-independent mechanisms. In *Eμ-Myc* lymphoma cells, it has been reported that low-dose APR-246 (15 μM) causes cell death by inducing the intrinsic pathway of apoptosis that is p53-dependent, while high-dose APR-246 (50 μM) induces non-apoptotic, p53-independent cell lysis [21]. In contrast, our results indicate that cleaved caspase-3-mediated apoptosis is the major form of cell death induced by both 25 and 50 μM of APR-246 in Molm13 AML cells, underlining a possible greater dosage window for AML where APR-246 would remain selective and is clinically relevant [32] (Figure 2). In addition, APR-246 significantly induces *PUMA* and *NOXA*, both of which are transcriptional targets of p53 that promote apoptosis, in *TP53* WT Molm13 and MV4-11 cells. Still, it has been shown that extremely high doses of APR-246 (50 to 60 mM), which are about 1000-fold higher than those we used in this study, can induce cleaved caspase-3-independent ferroptosis in multiple human AML cell lines in vitro [18]. Apoptosis-independent cell death, such as necroptosis and ferroptosis, also contributes to APR-246-induced cell death in certain solid tumor cell lines in vitro [21]. Together, these findings indicate that APR-246 can induce diverse forms of cell death that, at least partially, depend on treatment concentration, cell type, and p53 functional status.

Although APR-246 is intended to reactivate mutant p53 proteins, it also increases ROS accumulation by affecting the GSH synthesis pathway [18,19,20]. We corroborate this data in our observation that APR-246 increases cellular ROS in Molm13 cells in a dose-dependent manner, as treatment of Molm13 cells with NAC completely abrogates the cell toxicity induced by APR-246. Notably, loss of p53 significantly decreases ROS accumulation induced by APR-246 and prevents the induction of NRF2 target genes upon APR-246 treatment (Figure 3). These results suggest that the loss of functional p53 protects cells from APR-246 by reducing ROS accumulation. Previous studies have demonstrated that p53 can transcriptionally regulate multiple redox-related genes and promote mitochondrial ROS generation during apoptosis [45]. In addition, p53-mediated induction of pro-apoptotic targets such as PUMA and NOXA may further enhance mitochondrial dysfunction and oxidative stress [46]. Therefore, WT p53 may amplify APR-246-induced ROS accumulation through both transcriptional regulation of antioxidant/redox pathways and mitochondrial apoptotic signaling. Without p53, ROS levels induced by APR-246 do not accumulate in great enough amounts to activate the NRF2 antioxidant pathway in Molm13 cells treated with 25 μM of APR-246 and MV4-11 cells treated with 10 μM of APR-246. These findings indicate that functional p53 holds a crucial role in the dual-acting mechanism of APR-246 by responding to and amplifying APR-246-induced ROS accumulation in a positive feedback loop. In fact, it has been shown that the transcriptional induction of redox-related genes and the formation of ROS are key mechanisms by which p53 induces apoptosis [45,47]. In multiple cell types, apoptosis induced by p53 overexpression is ROS dependent. For example, overexpression of p53 in a colorectal cancer (CRC) cell line DLD1 significantly induces multiple genes that are involved in the production of ROS or the cellular response to ROS [45]. With this, it is likely that p53, whether restored to functional status by APR-246 or intrinsically WT, is critical to promote the oxidative stress induced by the drug and enhance it to initiate caspase-3-mediated cell death. Thus, our findings reveal a pivotal role of WT p53 protein in facilitating and amplifying an ROS-mediated cell death induced by APR-246 in *TP53* WT AML cells and normal HSPCs.

Recently, a Phase III clinical trial that combined APR-246 with azacitidine versus azacitidine alone in patients with *TP53*-mutant myelodysplastic syndromes (MDS) (NCT03745716) failed to meet the predefined efficacy criterion [33]. However, in this trial, the criteria of a “*TP53*-mutated MDS” were broadly defined, with no stratification between the different types of mutations for the cancer in the patients studied, with the only requirement being “*TP53* frameshift, nonsense, or missense mutations.” The results from our study using isogenic AML cells suggest that myeloid neoplasm patients who harbor frameshift or nonsense mutations of *TP53*, which account for around 25% of *TP53*-mutant patients [4,6], may be substantially resistant to APR-246 compared with myeloid neoplasm patients who harbor missense mutations of *TP53.* Even though all our experiments were performed in vitro due to the unfavorable pharmacokinetic profile of APR-246, this remains a limitation of the current study. Future studies using in vivo leukemia models or patient-derived samples will be important to further validate the role of *TP53* status in regulating sensitivity to APR-246. Nevertheless, our findings remain informative and suggest that the *TP53* mutational status may influence therapeutic responses to APR-246 in clinical settings.

**Figure 5:**
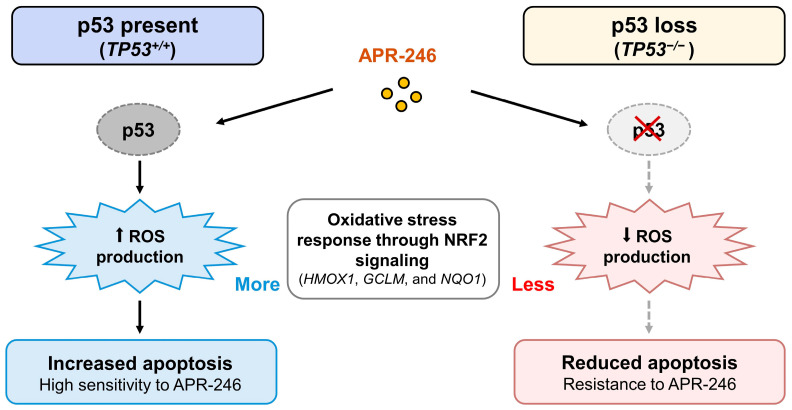
Schematic illustration of the proposed mechanism by which p53 modulates APR-246-induced ROS production and cellular response. Schematic representation of the role of p53 in regulating cellular sensitivity to APR-246. In *TP53*-proficient cells (*TP53^+/+^*), APR-246 treatment induces robust ROS production, which activates the NRF2 antioxidant response pathway, including downstream target genes such as *HMOX1*, *GCLM*, and *NQO1*. Concurrently, p53 further enhances oxidative stress and induces pro-apoptotic targets such as PUMA and NOXA, thereby promoting apoptotic cell death. In contrast, loss of *TP53* (*TP53^−/−^*) attenuates APR-246-induced ROS accumulation, resulting in weaker activation of the NRF2-mediated antioxidant response and reduced apoptotic signaling, ultimately leading to decreased cellular sensitivity to APR-246.

Notably, our findings that APR-246 markedly suppresses the clonogenicity of normal HSPCs suggest that hematopoietic toxicity may represent a potential limitation of APR-246-based therapies. Because WT p53 may also enhance APR-246-induced ROS accumulation and apoptosis in normal hematopoietic progenitors, careful optimization of treatment dose and scheduling may be necessary to minimize bone marrow toxicity in future clinical applications. In summary, our results reveal that, in at least a subset of AML cell lines and normal HSPCs, APR-246-induced ROS production and cytotoxicity are significantly enhanced in the presence of WT p53 protein (Figure 5). These findings suggest that APR-246 may be a promising therapeutic approach to treat *TP53* WT AML patients.

## 4. Materials and Methods

### 4.1. Cell Culture and Cellularity Assay

AML cells were cultured in RPMI 1640 culture media (Gibco, Waltham, MA, USA: 11875093) supplemented with 1 mM Sodium Pyruvate (Gibco: 11360070), 10% Fetal Bovine Serum (Gibco: A5669801), and Anti/Anti (Gibco: 15240062). Cells were maintained at a density between 2.5 × 10^5^ and 2 × 10^6^ cells/mL in 10 cm culture dishes while being incubated at 37 °C. For assays, AML cells were plated in clear 96-well plates at a density of 4 × 10^4^ cells/well, suspended in 200 μL of media. APR-246 (provided by Aprea Therapeutics) resuspended in MES (Boston Bioproducts, Milford, MA, USA: BB-112) was added to these wells as they were plated at final concentrations between 1 and 100 μM. Three wells containing only culture media were added to each plate to act as blanks during analysis. To assess the cellularity of AML cells, 20 μL of a 500 μg/mL solution of Resazurin (ThermoFisher Scientific, Waltham, MA, USA: B21187.03) resuspended in sterile PBS (Gibco: 10010049) was added to each well before a 4hr incubation at 37 °C. These plates were then read on a plate reader with an excitation/emission of 530/590 nm.

### 4.2. RNA Isolation and cDNA Synthesis

AML cells were plated at a density of 2 × 10^5^ cells/mL in a 6-well plate containing 3mL of media per well. These cells were then treated with either MES or APR-246 after 6 h. Cells were then collected and centrifuged at 300× *g* for 5 min before resuspending in 1 mL of cold, sterile PBS. The cell suspension was then centrifuged again at 400× *g* for 5 min before removing the supernatant. The cell pellets were snap frozen in liquid nitrogen and stored at −80 °C for RNA or protein isolation. Snap frozen cell pellets were resuspended in 800 μL of TRIzol reagent (ThermoFisher: 15596026) before the addition of 160 μL of BCP Reagent (Catalogue # BP 151). This mixture was then centrifuged at 12,000× *g* for 15 min at 4 °C. 350 μL of the aqueous supernatant was removed and mixed with 350 μL of 100% RNAase-free EtOH (Catalogue # 44-505-00ML). This solution was then processed following the instructions provided by the RNA Clean and Concentrator kit (Zymo Research, Irvine, CA, USA: R1013). The resulting isolated RNA was then analyzed for purity and concentration via a spectrophotometer. Subsequently, 1000 ng of quantified RNA was then used to synthesize cDNA according to the instructions for the iScript cDNA Synthesis Kit (BioRad, Hercules, CA, USA: 1708891).

### 4.3. RT-qPCR Analysis

cDNA synthesized from AML cells was used for qPCR according to the manufacturer’s instructions for Power SYBR Green PCR Master Mix (ThermoFisher: 4367659) prior to qPCR analysis on an Applied Biosystems QuantStudio 6 Flex machine (ThermoFisher: 4485697) via the Fast 96W ddCT SYBR setting. qPCR data were analyzed using the ddCT method on the QuantStudio Design and Analysis software (v1.7.2).
Target GenesHumanForward SequenceReverse Sequence*PUMA*ATGGCGGACGACCTCAACAGTCCCATGAAGAGATTGTACATGAC*NOXA*ACCAAGCCGGATTTGCGATTACTTGCACTTGTTCCTCGTGG*TP53*CACATGACGGAGGTTGTGAGACACGCAAATTTCCTTCCAC*HMOX1*AAGACTGCGTTCCTGCTCAACAAAGCCCTACAGCAACTGTCG*GCLM*CATTTACAGCCTTACTGGGAGGATGCAGTCAAATCTGGTGGCA*NQO1*GAAGAGCACTGATCGTACTGGCGGATACTGAAAGTTCGCAGGG*TBP*TGCACAGGAGCCAAGAGTGAACACATCACAGCTCCCCAC CA

### 4.4. Flow Cytometry

Cells destined for apoptosis analysis were plated at 2 × 10^5^ cells/mL in a 6-well plate. These cells were treated with MES, 25 μM, or 50 μM of APR-246 for 16 h before pelleting. Samples were then treated with the FLICA Casp3/7 assay (ImmunoChemistry Technologies, Davis, CA, USA: 9125) according to the manufacturer’s instructions. These samples were then analyzed on a BD FACS Canto B. Cells intended for ROS activity analysis were also plated at 2 × 10^5^ cells/mL in a 6-well plate. These cells were treated with MES, 25 μM or 50 μM of APR-246 for 6 h before treatment with 2′,7′-dichlorodihydrofluorescein diacetate for 30 min. Cells were then pelleted at 300× *g* for 5 min and resuspended in PBS before another pelleting step and resuspension in Flow Buffer (HBSS with Ca^2+^ and Mg^2+^, 5% fetal bovine serum, 2 mM EDTA). These samples were then analyzed on a BD FACS Canto B. All flow cytometry data were analyzed using FlowJo (v10).

### 4.5. Generation of Stable TP53 shRNA-Expressing AML Cells

Human AML cell lines (OCI-AML2 and MV4-11) were obtained from the Duke Life Science Facility, mycoplasma tested, and identities verified by short tandem repeat analysis before expanding and stocking. Mycoplasma status was re-verified after stable line generation. Plasmids bearing human *TP53*-directed shRNA were a kind gift of Lina Benajiba (INSERM Institute of Health and Medical Research, Paris, France). Briefly, shRNA sequences targeting human *TP53* transcript or nontargeting scramble shRNA sequences were cloned into the SGEN plasmid (Addgene 11171, kind gift of Johannes Zuber) using XhoI and EcoRI restriction sites. The shRNA sequences were as follows: control shRNA, TAGATAAGCATTATAATTCCTA; *TP53* shRNA, TATACAAGAGATGAAATCCTCC. Lentiviral particles were generated by co-transfecting the SGEN-shRNA plasmid with pMD2.G and psPAX2 (Addgene plasmids 12259 and 12260, kind gift of Didier Trono). Stable cell lines were generated by coincubating the lentiviral shRNA particles with the AML cell lines for 36 h in the presence of polybrene. At day 3 post-infection, G418 was administered at a concentration of 400 μg/mL for 7 days to select for cells with stable plasmid integration before verifying knockdown by RT-qPCR and the use of cell lines in experiments.

### 4.6. Isolation of CD34^+^ Cells from Human Cord Blood

This study was approved under Duke Health Institutional Review Board (IRB) protocol # ProAll units used for isolation of cord blood CD34^+^ cells were <48 h old. CD34^+^ cells were isolated using the CD34 Microbead Kit (Miltenyi Biotec, Bergisch Gladbach, Germany). Briefly, for positive selection, 1 × 10^8^ cells were resuspended in 300 μL of 10% FBS and incubated with 100 μL of FcR blocking buffer and 100 μL of CD34 microbeads (Miltenyi Biotec, 130-100-453) for 30 min in the refrigerator at 4 °C. Cells were washed with 10% FBS and centrifuged at 1400 rpm for 5 min. The cell pellet was resuspended in 500 μL of 10% FBS per 10^8^ cells and collected CD34^+^ cells using LS magnetic columns (Miltenyi Biotec: 130-042-401) according to the manufacturer’s protocol. The cell counts and the purity of CD34^+^ cells were determined by flow cytometric analysis.

### 4.7. Colony Formation of Mouse Bone Marrow-Derived HSPCs

All animal procedures for this study were approved by the Institutional Animal Care and Use Committee (IACUC) at Duke University (protocol number A215-20-11 was approved on 9 November 2020). All methods were performed in accordance with the relevant guidelines and regulations. The study is reported in accordance with ARRIVE guidelines. Whole bone marrow cells were harvested from Tie2-Cre; *Trp53^FL/+^*, Tie2-Cre; *Trp53^FL/−^*, Tie2-Cre, and Tie2-Cre; *LSL-Trp53^R172H^* mice according to the published methods [48]. All these mouse models were described previously [37,49]. Whole bone marrow cells were then plated at a density of 2 × 10^4^ cells/mL using a Methocult-based hematopoietic progenitor selection media (STEMCELL Technologies: #03434) according to the manufacturer’s instructions. These cells were then incubated at 37 °C for 5–7 (10–12) days before counting colonies consisting of >50 cells.

## Data Availability

The original contributions presented in this study are included in the article/Supplementary Materials. Further inquiries can be directed to the corresponding author.

## Supplementary Materials

The following supporting information can be downloaded at: https://www.mdpi.com/article/10.3390/ijms27114974/s1.

## Abbreviations

The following abbreviations are used in this manuscript:

HSPC	Hematopoietic stem/progenitor cells
AML	Acute myeloid leukemia
GSH	Thiol-rich glutathione
ROS	Reactive oxygen species
NAC	N-acetyl cysteine
WT	Wild type
