# Supplementary material for: Wild-Type p53 Protein Enhances APR-246-Induced Cytotoxicity in Acute Myeloid Leukemia and Normal Hematopoietic Stem/Progenitor Cells
Source: Int J Mol Sci. 2026 May 30;27(11):4974. doi: 10.3390/ijms27114974 (PMC13257158; doi:10.3390/ijms27114974)
Supplement: Supplementary file 1 [file ijms-27-04974-s001.zip › ijms-4200241-supplementary.pdf]

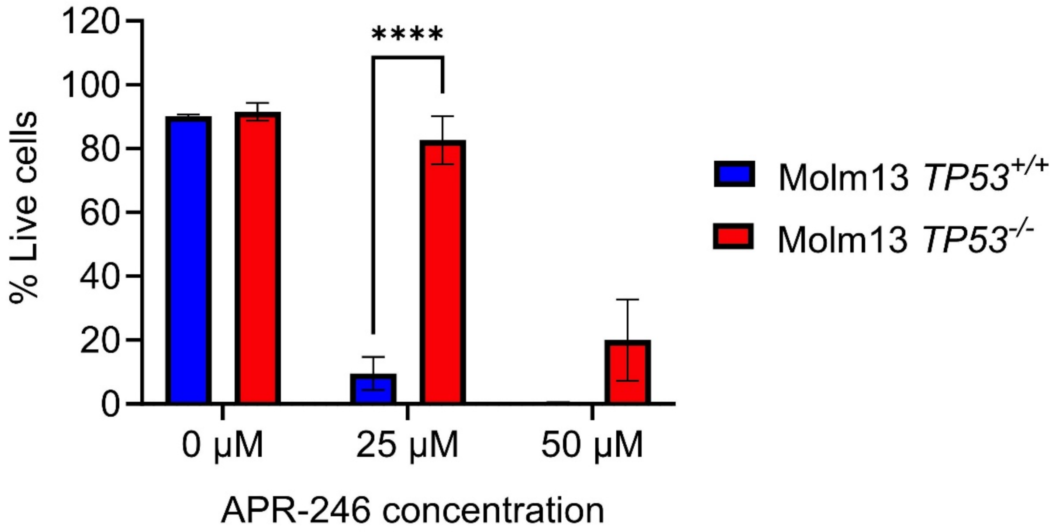

Figure S1. Loss of p53 reduces ARP-246-induced cell death of Molm13 AML in vitro. Representative flow cytometry plots of Molm13 *TP53*$^{+/+}$ and Molm13 *TP53*$^{-/-}$ cells treated with MES control (0 μM), 25 μM, and 50 μM APR-246 for 16 hours in vitro. Fresh cells were stained with the FLICA Caspase-3/7 Assay Kit and propidium iodide (PI). Quantification of live cells that are double negative for cleaved caspase 3/7 and PI in Molm13 *TP53*$^{+/+}$ and Molm13 *TP53*$^{-/-}$ cells treated with MES control (0 μM), 25 μM, and 50 μM APR-246 for 16 hours in vitro. N=3 independent experiments. The data are presented as mean+/-SEM. ****$p<0.0001$ by two-way ANOVA with Bonferroni post hoc test.

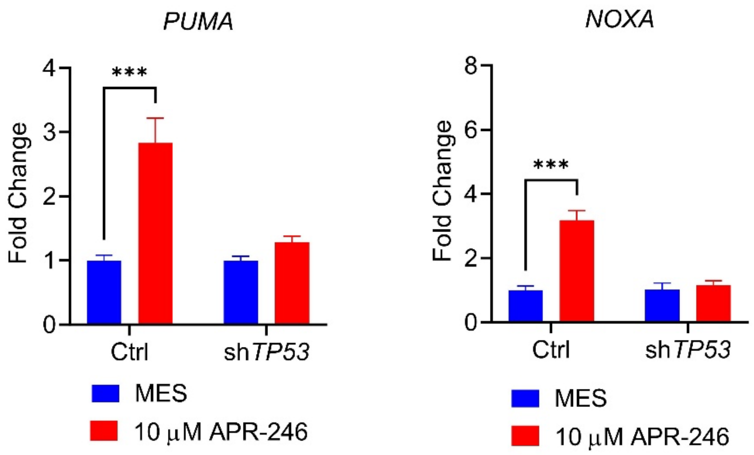

Figure S2. Knockdown of p53 suppresses APR-246-induced pro-apoptotic genes in MV4-11 cells in vitro. Examination of *PUMA* and *NOXA* mRNA in MV4-11 AML cell lines expressing control (Ctrl) shRNA or an anti-*TP53* shRNA (sh*TP53*) treated with MES control (0 μM) and 10 μM APR-246 for 6 hours in vitro. N=3 independent experiments. The data are presented as mean+/-SEM. ***p<0.001 by two-way ANOVA with Bonferroni post hoc test.

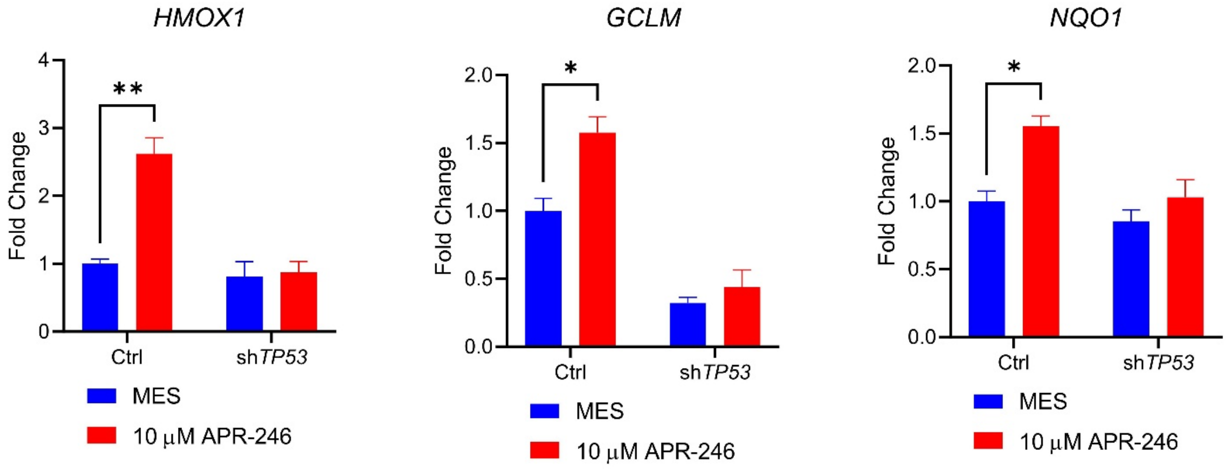

Figure S3. Knockdown of p53 suppresses APR-246-induced NRF2 target genes in MV4-11 cells in vitro. Examination of mRNA expression of NRF2 target genes, including *HMOX1*, *GCLM*, and *NQO1*, in MV4-11 AML cell lines expressing control shRNA (Ctrl) or an anti-*TP53* shRNA (sh*TP53*) treated with MES control (0 μM) and 10 μM APR-246 for 6 hours in vitro. N=3 independent experiments. The data are presented as mean+/-SEM. *$p<0.05$ **$p<0.01$ by two-way ANOVA with Bonferroni post hoc test.

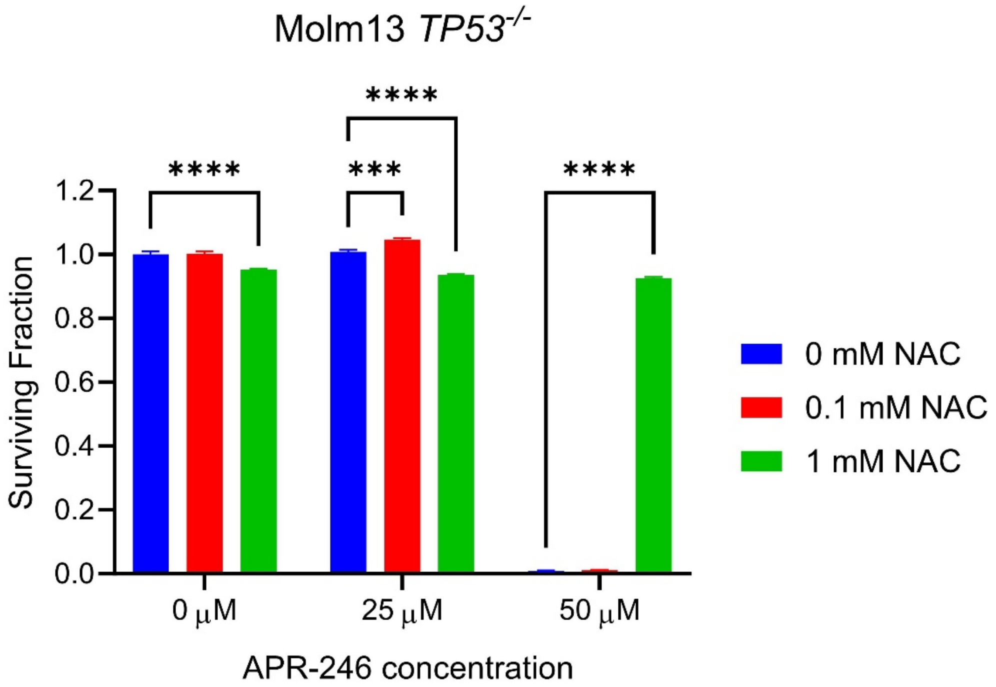

Figure S4. N-acetyl cysteine rescues APR-246-induced cytotoxicity in Molm13 *TP53*$^{-/-}$ cells. Molm13 *TP53*$^{-/-}$ cells were treated with MES control (0 μM), 10 μM, and 25 μM APR-246 for 72 hours in vitro. 0, 0.1, or 1 mM of N-acetyl cysteine (NAC) was added to the cells immediately after the initiation of APR-246 treatment. The surviving fraction was assessed using a resazurin assay. N=3 independent experiments. The data are presented as mean+/-SEM. ***p<0.001 ****p<0.0001 by two-way ANOVA with Bonferroni post hoc test.
